# A Physics-Guided Neural Network for Predicting Protein–Ligand Binding Free Energy: From Host–Guest Systems to the PDBbind Database †

**DOI:** 10.3390/biom12070919

**Published:** 2022-06-29

**Authors:** Sahar Cain, Ali Risheh, Negin Forouzesh

**Affiliations:** 1 Department of Computer Science, California State University, Los Angeles, CA 90032, USA; srohani3@calstatela.edu; 2 Department of Computer Engineering, Amirkabir University of Technology, Tehran 15914, Iran; ali.risheh@aut.ac.ir

**Keywords:** binding free energy, implicit solvent model, graph convolutional network

## Abstract

Calculation of protein–ligand binding affinity is a cornerstone of drug discovery. Classic implicit solvent models, which have been widely used to accomplish this task, lack accuracy compared to experimental references. Emerging data-driven models, on the other hand, are often accurate yet not fully interpretable and also likely to be overfitted. In this research, we explore the application of Theory-Guided Data Science in studying protein–ligand binding. A hybrid model is introduced by integrating Graph Convolutional Network (data-driven model) with the GBNSR6 implicit solvent (physics-based model). The proposed physics-data model is tested on a dataset of 368 complexes from the PDBbind refined set and 72 host–guest systems. Results demonstrate that the proposed Physics-Guided Neural Network can successfully improve the “accuracy” of the pure data-driven model. In addition, the “interpretability” and “transferability” of our model have boosted compared to the purely data-driven model. Further analyses include evaluating model robustness and understanding relationships between the physical features.

## 1. Introduction

Proteins perform biological functions through interaction with other biomolecules, including ligands that are (small) molecules capable of binding to a target protein, often with high affinity and specificity [1]. Protein–ligand interaction is central to several biological processes, e.g., cellular signal transduction, viral invasion, DNA replication, and cellular energy production [2]. It also has vast applications in the early stages of drug discovery [3]. One key component of protein–ligand interactions is the binding free energy change, ΔΔG, occurring between the protein and the ligand upon the ligand’s attachment. This physiochemical feature heavily dictates how strongly a protein and ligand interact and is particularly useful to understand for drug design [4]. While wet-lab experiments accurately estimate ΔΔG, they are significantly slow, costly, and laborious. On the other hand, computational simulations enable significantly faster estimation of ΔΔG and shed light on the binding mechanism of various structures [5].

A wide range of computational methods trades off between physical rigor and computational time for ΔΔG calculations. On one side of the spectrum, there is molecular docking [6,7] that provides on-the-fly determination of the best poses of a ligand based on different measurements, including ΔΔG. Such methods have vast usage in high-throughput virtual screening and lead optimization, where a quick ranking of candidate drugs is central. However, molecular docking fails to calculate ΔΔG accurately due to many rough approximations, e.g., receptor flexibility, strain energies, and various entropies. On the other side of the spectrum, there exist alchemical free energy methods which evaluate ratios of partition functions and, therefore, calculate entropic and enthalpic components of ΔΔG accurately [8,9,10]. The drawback, though, is the high computational cost needed to run such simulations. In the middle of the spectrum, there exist molecular mechanics Poisson–Boltzmann surface area (MM/PBSA) and molecular mechanics generalized Born surface area (MM/GBSA) [11,12,13,14] methods that balance the aforementioned accuracy vs. computational cost. MM/PB(GB)SA have been employed in drug design challenges [15], studying large benchmarks of protein–ligand complexes [16], and examining virus–receptor interactions [17,18]. A key factor determining the accuracy of these methods is the underlying implicit solvent.

Implicit solvent modeling is one of the most popular computational methods that consider solvent (usually water) as one continuum component [19]. Within this framework, the calculation of ΔΔG could be conducted more efficiently compared to explicit solvents [20,21]. Practical implicit solvent models have broad applications in computer-aided drug design, particularly in protein folding and molecular dynamics simulation [11]. Poisson–Boltzmann (PB) [22,23] and generalized Born (GB) [24,25,26] models are the two main classes of implicit solvent models that have been used widely in static and dynamic simulations of protein–ligand interactions [27,28]. Despite many years of research, there are serious concerns regarding the lack of accuracy in implicit solvent modeling [29] that is to some extent unavoidable due to the underlying physical approximations, such as the elimination of water response beyond the dipole and polar to non-polar coupling [30]. Recently, cutting-edge technology has provided opportunities to compensate for this issue.

With the ever-increasing determination of protein–ligand structures and the emergence of powerful hardware, machine learning (ML) techniques have been extensively utilized to identify structural patterns and predict binding profiles [31]. While these methods demonstrate promising results, two major concerns question their credibility: first and foremost, the sole dependence of ML on data raises criticism of overfitting and the lack of transferability, knowing that the available (experimental) data do not represent the entire system. This concern becomes more critical when the training and test sets are small; which is the case in many real-world applications, including drug discovery, where generating clean and accurately labeled data is costly and time-consuming. Secondly, the “black-box” characteristic of ML leads to uninterpretable results that, in many cases, do not agree with well-known physical models. Apparently, such models cannot be accepted in the scientific community even if they work perfectly accurately on the given data. What can bridge the gap between uninterpretable and potentially over-fitted data-driven models and relatively inaccurate theoretical models is a combination of the two known as Theory-Guided Data Science [32,33]. This new paradigm has shown accurate, interpretable, and transferrable results in modeling diverse scientific realms, including quantum chemistry [34], genetics [35], fluid mechanics [36], and material sciences [37,38,39].

The main objective of this work is to propose a transferable and interpretable model that can estimate ΔΔG more accurately compared to the reference physics-based model. Our approach is to incorporate experimental data into an implicit solvent so that, with adherence to the physical model, new features extracted from the input structures could improve the accuracy of ΔΔG calculations. The proposed hybrid model consists of two main components: a GB model called GBNSR6 [40] and a state-of-the-art neural network. Compared to other flavors of GB, GBNSR6 [40] calculates ΔΔG of protein–ligand complexes efficiently and accurately when the reference is explicit solvent models [41]. However, the ∼5 kcal/mol error compared to the standard TIP3P water model necessitates improved accuracy [42,43] (chemical accuracy: 1 kcal/mol). With that, the second component is designed based on Graph Convolutional Network, which has demonstrated remarkable performance in extracting spatial and structural features in different domains, including computational structural biology [44]. The proposed Physics-Guided Neural Network was first introduced in [45]. Here, we analyze and test it on a PDBbind (refined) set of a comprehensive collection of protein–ligand complexes and small host–guest systems. Results of the proposed hybrid model have been compared with reference experiments as well as pure data-driven and physics-based models.

## 2. Background

### 2.1. Physics-Based Model: GBNSR6

Accurate calculation of free energy is highly dependent on precise measurements of electrostatic interactions in an aqueous environment. To accomplish this, biomolecules are often placed in a solvent that is usually water accompanied by counter-ions to ensure a neutral environment. This model is called the explicit solvent model, where the water molecules are explicitly measured in calculating the electrostatic energy of the system. Although this method proved to be highly accurate, it suffers from substantial computational costs, especially for large structures. One alternative solution is implicit solvent modeling, where explicit water molecules are replaced with an infinite continuum medium that has equivalent dielectric properties as water [19].

Binding free energy (ΔΔG) of a molecular system is calculated as

(1) ΔΔG=ΔH−TΔS

where ΔH is the enthalpy change, ΔS is the entropy change of the system, and *T* is the temperature in Kelvin. Enthalpy is a property of a thermodynamic system and is defined as the sum of the system’s internal energy and the product of its pressure and volume. In the implicit solvent framework, ΔH is decomposed into the gas-phase molecular mechanics energy, $\Delta E_{MM}$, and solvation free energy, $\Delta G_{solv}$. $\Delta E_{MM}$ consists of the changes in internal energy, van der Waals energy, and the change in electrostatic energy. $\Delta G_{solv}$ consists of polar and nonpolar components. $\Delta G_{pol}$ (the largest component of the total energy for biomolecules) is calculated with either a PB or GB model. GB is chosen in this research since it has shown to be an efficient approximation of PB with quite accurate results [40]. A GB model with ALPB correction [46] has the following formula:

(2) $$\begin{array}{c} \begin{array}{c} \Delta G_{pol}\approx -\frac{1}{2}\left(\frac{1}{\epsilon_{in}}-\frac{1}{\epsilon_{out}}\right)\frac{1}{1+\beta \alpha }\sum_{ij}q_{i}q_{j}\left(\frac{1}{f_{ij}^{GB}}+\frac{\alpha \beta }{A}\right) \end{array} \end{array}$$

where $\epsilon_{in}$ and $\epsilon_{out}$ are the dielectric constants of the solute and the solvent, respectively, $\beta =\epsilon_{in}/\epsilon_{out}$, α=0.571412, and *A* is the electrostatic size of the molecule, which is essentially the overall size of the structure which can be computed analytically [46]. We employ the most widely used functional form [47] of $f_{ij}^{GB}$: $f_{ij}^{GB}={\left[r_{ij}^{2}+R_{i}R_{j}\exp(-r_{ij}^{2}/4R_{i}R_{j})\right]}^{\frac{1}{2}}$, where $r_{ij}$ is the distance between atomic charges $q_{i}$ and $q_{j}$, and $R_{i}$, $R_{j}$ are the so-called *effective Born radii* of atoms *i* and *j*, which represent each atom’s degree of burial within the solute. In GBNSR6, effective born radii are calculated with “R6” equation [40]. The grid-based implementation of GBNSR6 is freely available in the AMBER suite of biomolecular simulation programs [48].

Entropy is a measure of the molecular disorder or randomness of a system. It is associated with conformational energy loss when a free-state ligand binds to the corresponding unbound-state receptor. Standard methods for estimating entropic component are normal mode analysis (NMA) [49] and quasi-harmonic approximation [50]. Due to its computational complexity, though, the entropy calculation in many studies of free energy is ignored. In this study, entropy is estimated as a new feature by subtracting the enthalpy calculated by the physics-based component from the experimental reference values. See Materials and Methods for more information.

### 2.2. Data-Driven Model: GCN

Graph convolutional network (GCN) is a specialized convolutional neural network that accepts graphs as input and applies several layers of filters to learn patterns [51,52]. Particularly, GCN takes as input a feature description $x_{i}$ for every node *i* summarized in an N×D feature matrix X, where *N* is the number of nodes and *D* is the input features. In addition, a representative description of the graph structure in matrix form, which is typically in the form of an adjacency matrix A. The output is a node-level matrix Z that is an N×F feature matrix, where *F* is the number of output features per node. Graph-level output, *z*, can be modeled by introducing some form of pooling operation. With that, every neural network layer is written as a nonlinear propagation function $H^{\left(l+1\right)}=f\left(H^{\left(l\right)},A\right)$ with $H^{\left(0\right)}=X$ and $H^{\left(L\right)}=Z$ (or *z* for graph-level outputs), *L* being the number of layers. The specific models then differ only in how f(.,.) is chosen and parameterized.

In this work, two GCNs available in Deepchem [31,53] are employed: GraphConv model and AtomicConv model. GraphConv model implements the graph convolutional model presented in [54]. These graph convolutions start with a per-atom set of descriptors for each atom in a molecule, then combine and recombine the descriptors over convolutional layers. AtomicConv model functions as a variant of graph convolution [31]. The difference is that the “graph” in this model is the nearest neighbors graph in 3D space. The AtomicConv model leverages these connections in 3D space to train models that learn to predict energetic states starting from the spatial geometry of the model. These two models have been utilized as the reference data-driven models in comparison with our hybrid model. Due to its flexibility, the GraphConv model has been chosen as the GCN component in the proposed PGNN model.

## 3. Materials and Methods

### 3.1. Featurization and Parameterization

Model training is performed with a combination of structure-based and physics-based features. Structure-based features are directly extracted from PDB files. ConvMolFeaturizer [31] from Deepchem is employed to represent atom features in the form of a graph which is an implementation of Duvenaud graph convolutions [54]. Every protein–ligand complex is featurized based on each atom’s neighborhood and is transformed to a 2D matrix, $X_{N\times D}$, where *N* is the number of atoms in a complex and *D* is the number of features for each atom. ConvMolFeaturizer extracts 75 features for each atom that is a binary representation of atom type, atom hybridization type, implicit valence, aromaticity, atom degree, number of hydrogens, number of radical electrons, and formal charges of each atom.

Physics-based features, **P**, of each molecule are calculated using GBNSR6 available in Ambertools 2020 [55]. In short, the GBNSR6 model is executed on the complex, protein, and ligand structures. Electrostatic energy (EELEC), electrostatic energy for 1–4 bonded atoms (1–4-eel), non-polar solvation energy (ESURF), polar solvation energy (EGB), and Van der Waals (VDWAALS) energy are extracted accordingly. (PBSA [56,57] model is employed to calculate Van der Waals energy since this calculation is not implemented in GBNSR6). Total enthalpy is subtracted from the experimental ΔΔG values to account for entropy estimation. This number is added to the model as the last physics-based feature. See Table 1 for more details.

### 3.2. Hybrid Model: PGNN

**Figure 1:**
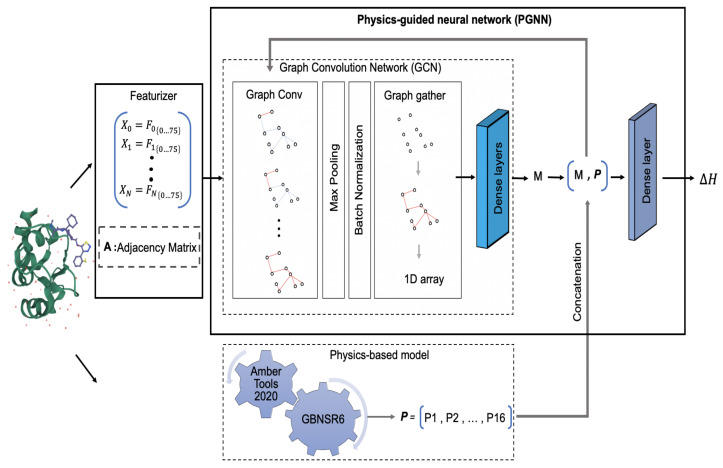
Workflow of the proposed PGNN model. The sample input structure, the complex of the BIR domain of MLIAP and GDC0152, is selected from the PDBbind refined set [58]. After featurization, the structure-based features along with the adjacency matrix **A** enter the network. The results of the first network iteration are the model variable *M*. The output of the physics-based model is vector **P**, which is concatenated with *M*. The resulting vector goes through several iterations before entering the final dense layer.

The proposed Physics-Guided Neural Networks (PGNN) is a GCN with integrated physics-based features. The architecture of this model is shown in Figure 1. The model employs a GCN [31] to capture spatial features of the structures in the 3D space. The PGNN model consists of a couple of GraphConv layers of fixed channel size (training epoch: 100, learning rate: 0.001). The activation function used for GraphConv layers is the Tanh function to provide output in the continuous range of [−1,1]. This layer combines the features of each atom with ten nearest neighbor atoms and creates a new feature vector for each atom. The output of GraphConv is a matrix of order N×75×10. A batch normalization layer is applied to improve the learning process, followed by a single-dimensional max pooling to minimize the feature space. Finally, the GraphGather layer is used to combine the data from all different nodes. The output of this layer is the model variable *M* that later concatenates with vector **P** containing physics-based features. The new vector, (M,P), is fed to the final dense layer to estimate the ΔΔG. The activation function used in the last two dense layers is Relu. The loss function is designed to minimize the empirical error or, in other words, to minimize the RMSE in calculating ΔΔG compared to the experiments.

The following thermodynamic equation is integrated into the learning process of the proposed PGNN model by initializing the weights of ΔH to 1, −1, −1 for the complex, protein and ligand, and −1 for entropy:

(3) $$\Delta \Delta G=[\Delta H_{complex}-\left(\Delta H_{ligand}+\Delta H_{protein}\right)]-T\Delta S$$

### 3.3. Datasets

**Figure 2:**
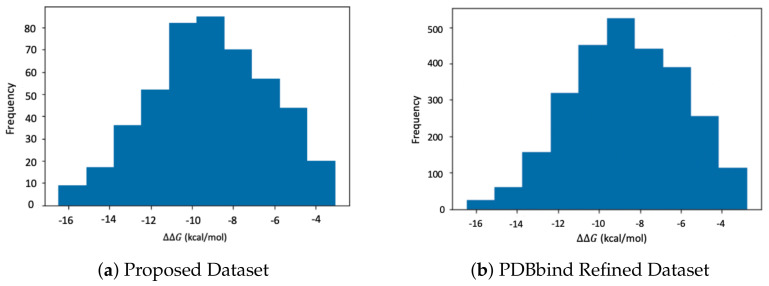
Distribution of ΔΔG values of PDBbind refined set vs. our sample dataset.

**PDBbind Refined Set v.2015.** The primary dataset used in this work is acquired from PDBbind refined set v.2015 [58]. This dataset is a comprehensive collection of experimentally measured ΔΔG values for 3706 protein–ligand complexes. However, due to the limited scalability of the DeepChem featurizer, a subset of 368 complexes of various sizes were picked for training and testing the proposed model. As illustrated in Figure 2, a subset has been selected with reference to the ΔΔG values of the original PDBbind dataset to ensure a comprehensive sampling. The size of the protein–ligand complexes in the sample dataset varies between 1200 to 43,000 atoms, which replicates the wide range of structure sizes in the original dataset. Extensive structure optimization and force field assignments are carried out using the protocol explained in [59]. Water molecules are eliminated from the structural (PQR) files using the CPPTRAJ library in Ambertools 2020. ANTECHAMBER program is used to generate custom residue topology files (prep files) using the general Amber force field (GAFF) [60] for ligands and FF14SB [61,62] for protein structures. The TLEAP program is employed to create the coordinate and topology files, and these files are then used to run GBNSR6. The dataset was split into training and test sets with a ratio of 3:1. Model training and testing were performed on San Diego Supercomputing Center (SDSC) clusters with 20 CPU cores and 242 GB of memory which took approximately 40 min.

**Host–Guest Systems.** Another dataset used in this work is a collection of small structures acquired from the host–guest benchmark [4] and SAMPL challenges [63]. This dataset consist of seven distinct hosts named Octa Acid (OA), tetra-endomethyl octa acid (TEMOA), Alpha-Cyclodextrin (aCD) and Beta-Cyclodextin (bCD) which are from the host–guest benchmark system [4], OctaAcidH (OAH) [64] and OctaAcidMe (OAMe) [65] from SAMPL5 challenge and Cucurbit[8]uril (CB8) from the SAMPL6 challenge [66]. The hosts are small molecules containing less than 100 atoms. These hosts bind their guests the same way proteins bind their ligands, so they can be considered as simple test cases for computational models of noncovalent binding. In addition, their small size and, in many cases, their rigidity makes it feasible to efficiently estimate ΔΔG values. Several guests are provided for each host, comprising 72 rigid complexes in total. The raw PDB files and pre-processed topology and coordinate files of the host–guest benchmark are freely available at https://github.com/mobleylab/benchmarksets (accessed on 7 April 2017). SAMPL5 and SAMPL6 structure files are taken from SAMPL Challenges GitHub repository. The enthalpic and entropic components of ΔΔG for these molecules are experimentally measured. Complex topology and coordinate files were further processed to strip water molecules and counterions and then split into host and guest using CPPTRAJ library on Ambertools 2020. The topology and coordinate files of hosts, guests, and complexes were then used to run GBNSR6. The datasets were split into training and test sets with a ratio of 3:1.

## 4. Results and Discussion

### 4.1. Accuracy of the PGNN Model

This section examines the accuracy of the proposed PGNN model compared to the two data-driven models: GraphConv model and AtomicConv model. It should be noted that calculating total ΔΔG through the selected implicit solvent is not possible since GBSNR6 merely accounts for the enthalpic component. Since enthalpic and entropic values have not been measured separately in the PDBbind refined set, the accuracy of the PGNN model is just compared with the data-driven models. Both of these models have been trained for 100 epochs with 4-fold cross-validation, which leads to 276 data points for training and 92 data points for validation and testing. The mean squared error (MSE) is defined as the regression loss function for the learning process of the two models. RMSE is used to measure the accuracy of each model; see Table 2.

The following conclusions can be drawn from Table 2: First, the GraphConv model outperforms the PGNN model on the training set but fails to do so on the test set. This observation implies overfitting of GraphConv on the training set. It also specifies that the physics-based features in PGNN played an essential role in guiding the neural network on unseen data. The AtomicConv model performs significantly better on the test set than on the training set. The inconsistency in estimating ΔΔG values on the two sets, though, raises concern about the transferability of this model. PGNN, on the other hand, shows more accurate results consistently on the training and test sets.

**Figure 3:**
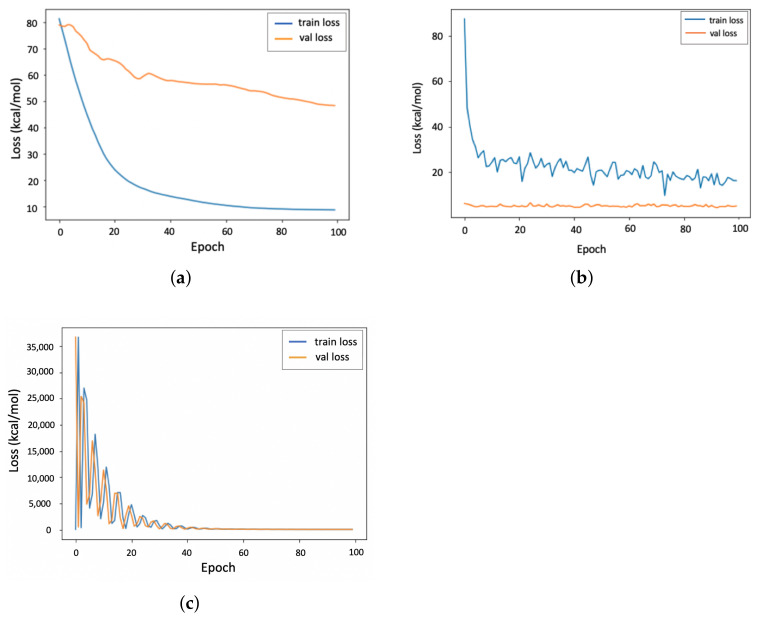
Validation loss (val loss) and training loss (train loss) per epoch for GraphConv, AtomicConv (data-driven) and PGNN (hybrid) models on the PDBbind dataset. (**a**) GraphConv model; (**b**) AtomicConv model; (**c**) PGNN model.

The average loss per epoch of the 4-fold cross-validation is illustrated in Figure 3: Figure 3a shows that the GraphConv model converges more quickly than the PGNN model, with a significant gap between the training and validation results. This observation, again, implies the overfitting of this data-driven model during the training process. Despite applying the Early Stopping rule in a different scenario (not demonstrated here), this gap never closed. Figure 3b demonstrates the poor performance of the AtomicConv model on the training set and a large gap between the training loss and the validation loss, which does not close after 100 epochs. In Figure 3c, the PGNN model shows more fluctuations throughout the learning process, indicating that the model can explore the solution space more effectively and learn the relation between the features more accurately. The model is more successful than the data-driven models in closing the gap between training and validation loss and minimizing the error. This observation demonstrates that the PGNN model is less likely to be overfitted on the training set and can predict the ΔΔG more accurately on unseen datasets.

### 4.2. Transferability of the PGNN Model

To evaluate the transferability of the proposed model, in addition to the PDBbind dataset, PGNN was trained and tested on the host–guest systems. According to Table 3, compared to GraphConv, PGNN is slightly less accurate on the training set but more accurate on the test set. Aligned with results in Table 2, this observation implies overfitting of the GraphConv model. GBNSR6 was utilized for calculating enthalpic values of binding free energy. Entropic values were borrowed from the experiment to account for total ΔΔG values. Since host–guest systems are small and rigid, this strategy does not significantly affect the accuracy of calculations. It can be seen that the physics-based model has poor performance in comparison to the other two models. This inaccuracy is the main motivation for proposing the PGNN model, which estimates ΔΔG values of the complexes about 6 kcal/mol more accurately. It should be noted that the AtomicConv model was not tested on this dataset since the input PDBbind dataset is hard coded in this model and could not be replaced with the host–guest systems.

**Figure 4:**
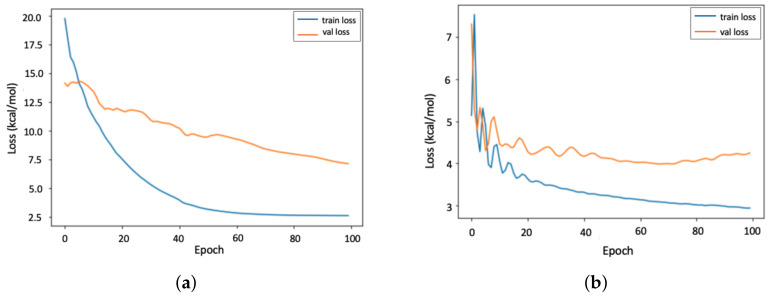
Validation loss (val loss) and training loss (train loss) per epoch for the GraphConv model (data-driven) and the PGNN model (hybrid) on the host–guest dataset. (**a**) GraphConv model; (**b**) PGNN model.

The average loss per epoch of the 4-fold cross-validation is illustrated in Figure 4. Similar to Figure 3, it is observed that GraphConv model (Figure 4a) converges more quickly than the PGNN model (Figure 4b) with a significantly larger gap between the training and validation results, which implies overfitting.

### 4.3. Interpretability of the PGNN Model

Calculating the binding free energy of biomolecules is a physics-based problem by nature. Therefore, it is crucial to interpret the relevant ML models by analyzing the model parameters compared to the physical laws. In the GraphConv model, the sum of the binary (atomic) features is directly related to ΔΔG values. However, the exact relation between these features and binding free energy is not straightforward due to the complicated architecture of the neural network. The proposed PGNN model utilizes physics-based features to learn the relationship between structural features. These features are used to analyze the interpretability of the proposed model.

According to Equation (3), physics-based features of the complex have positive coefficients, physics-based features of the protein and the ligand have negative coefficients, and entropy has a negative coefficient. Accordingly, we initialized the weights of complex parameters to +1, protein, ligand, and entropy parameters to −1, and the model variable to 0.5. It is shown in Table 4 that the model has retained the thermodynamic equation (i.e., Equation (3)) since the coefficients of the physics-based features are almost the same as before and after training. In addition, the coefficient of the model variable, which has been derived from binary features, has decreased from 0.5 to 0.45. In other words, the presented PGNN model has decreased the weight of the model variable in order to converge to a smaller gap between the predicted and actual values of ΔΔG.

### 4.4. Robustness Analysis

**Figure 5:**
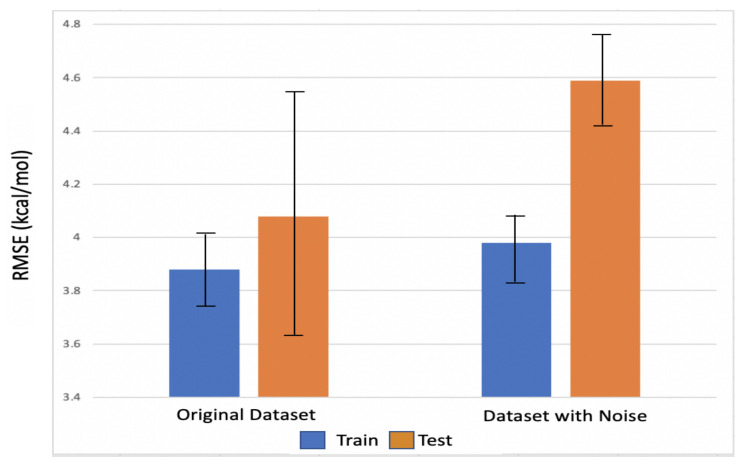
Error in calculating ΔΔG values on data with added noise compared to the original data.

Noise injection [67] is a standard practice in machine learning to study the robustness of a model. This technique can also be used in the training phase of a neural network when adding noise prevents memorizing the training samples and leads to a more robust model with lower generalization error. In this study, we tested the robustness of the PGNN model when it was trained and tested on the original dataset and the one with noise. Entropy was selected for robustness analysis since GBNSR6 does not calculate it directly. Instead, entropy values are given to the model as a difference between the experimental ΔΔG values and the enthalpic component calculated by GBSNR6. Gaussian noise, $N(\mu ,\;\sigma^{2})$, was added to the entropy feature such that μ represents the mean of entropic values over the entire dataset, and σ shows the standard deviation. According to Figure 5, it is observed that the accuracy of the model changes only 0.1 kcal/mol in the (noisy) training set and 0.5 kcal/mol in the (noisy) test set. These small changes in the RMSE of ΔΔG values confirm that the PGNN model is robust against small noises in entropy. Therefore, it is concluded that accurate entropy values, i.e., those calculated with NMA or Quasi-harmonic approximation, will not significantly affect the final results.

### 4.5. Feature Analysis

**Figure 6:**
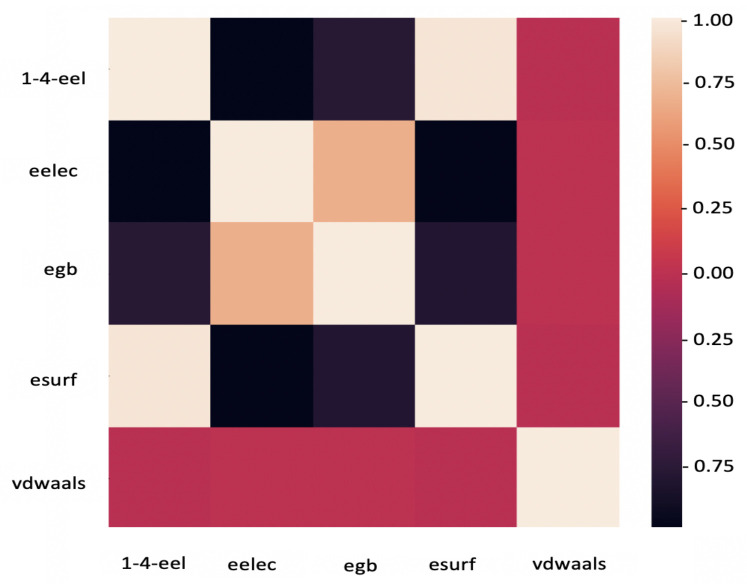
Correlation heatmap between the physics–based features of PDBbind structures.

The correlation heatmap between the physics-based features of protein–ligand complexes in the PDBbind dataset is shown in Figure 6. This figure provides insight into the relationship between the selected features, mainly whether they could be grouped or eliminated to avoid redundancy. It is demonstrated that van der Waals energy (vdwaals) has no linear relationship with other features. Therefore, keeping this feature as an independent indicator of short-range non-covalent energy is essential. The other features, though, show strong correlations: polar (egb) and non-polar (esurf) free energy components are negatively correlated. For future studies, it is worth merging the two and considering the total solvation free energy as a new feature. In addition, electrostatic energy for 1–4 bonded atoms (1–4-eel) strongly correlates with all the features, except for vdwaals. It is recommended to eliminate this feature and focus on other atomic interactions.

## 5. Conclusions

In this study, we have proposed a hybrid data-physics model to estimate the binding free energy of protein–ligand complexes with a wide range of structure sizes. This novel model inherits “interpretability” and “transferability” from the underlying physics-based model and “accuracy” from the data-driven model. As the first step, we have trained and tested the model on 368 protein–ligand structures selected from the PDBbind refined set and 72 host–guest systems. Results show that the combination of structural features extracted by the GraphConv model and physics-based features calculated by GBSNR6 enables the model to predict the binding free energy more accurately than the two data-driven models, i.e., the GraphConv model and the AtomicConv model. Compared to these models, the PGNN model consistently performs better on both training and test sets, reflecting its transferability between different datasets. Furthermore, analyzing the coefficients of physics-based features before and after training makes it possible to compare the results with physical laws and interpret them accordingly.

The robustness of the PGNN model has been evaluated by adding a noise function to a selected feature. Similar results before and after noise injection ascertain the robustness of the model. A closer look at the physical features and their relations demonstrates that van der Waals energy is the only one that does not correlate with other features. While it is recommended to keep this feature, eliminating or grouping other features should be tested in future works. The main objective of this paper was to introduce physics-data hybrid models and the corresponding neural network architecture. Extensive testing of this model on larger datasets, including new versions of PDBbind, is our immediate next step. Further extension of this work will be conducted by running short molecular dynamics (MD) simulations embedded in MM/GBSA to bring the dynamics of protein–ligand interactions into account.

## Figures and Tables

**Table 1:** Physics-based features calculated for complex, protein and ligand structures using GBNSR6.

Parameter	Description	Count
1–4-eel	1–4 Electrostatic energy	3
VDWAALS	Van der Waals energy	3
EELEC	Electrostatic energy	3
ESURF	Non-polar solvation energy	3
EGB	Polar solvation energy	3
Entropy	Entropy	1
Total		16

**Table 2:** Error in calculating ΔΔG values of the GraphConv model (data-driven), the AtomicConv model (data-driven), and the PGNN model (hybrid) on the PDBbind dataset. RMSE values are in kcal/mol.

	GraphConv	AtomicConv	PGNN
Training set	2.93 ± 0.08	16.37 ± 8.3	3.88 ± 0.13
Test set	6.90 ± 0.86	5.23 ± 0.40	4.08 ± 0.46

**Table 3:** Error in calculating ΔΔG values of the GraphConv model (data-driven), the PGNN model (hybrid), and the GBRNSR6 model (physics-based) on the host–guest dataset. RMSE values are in kcal/mol.

	GraphConv	PGNN	GBNSR6
Training set	1.61 ± 0.10	1.71 ± 0.11	8.22 ± 0.11
Test set	2.43 ± 1.27	2.05 ± 0.27	8.35 ± 0.34

**Table 4:** Coefficients of the physics-based features and the model variable in the last dense layer before and after training.

Physics-Based Parameters	Before Training	After Training
VDWAALS	−1,−1,1	−1.01,−0.99,0.99
EELEC	−1,−1,1	−0.99,−1.00,1.00
ESURF	−1,−1,1	−0.99,−0.99,1.00
EGB	−1,−1,1	−0.99,−1.00,1.00
1–4-eel	−1,−1,1	−0.99,−1.00,1.00
Entropy	−1	−0.99
Model variable	0.5	0.45

## Data Availability

The complete code repository, dataset, and scripts are publicly available at https://github.com/saharctech/Binding-Free-Energy-Prediction-PDBBind_REFINED_Dataset (accessed on 1 June 2022).

## References

[1] Du X., Li Y., Xia Y.L., Ai S.M., Liang J., Sang P., Ji X.L., Liu S.Q. (2016). Insights into protein–ligand interactions: Mechanisms, models, and methods. Int. J. Mol. Sci..

[2] Woo H.J., Roux B. (2005). Calculation of absolute protein–ligand binding free energy from computer simulations. Proc. Natl. Acad. Sci. USA.

[3] Jorgensen W.L. (2004). The Many Roles of Computation in Drug Discovery. Science.

[4] Mobley D.L., Gilson M.K. (2017). Predicting binding free energies: Frontiers and benchmarks. Annu. Rev. Biophys..

[5] de Ruiter A., Oostenbrink C. (2020). Advances in the calculation of binding free energies. Curr. Opin. Struct. Biol..

[6] Trott O., Olson A.J. (2010). AutoDock Vina: Improving the speed and accuracy of docking with a new scoring function, efficient optimization, and multithreading. J. Comput. Chem..

[7] Allen W.J., Balius T.E., Mukherjee S., Brozell S.R., Moustakas D.T., Lang P.T., Case D.A., Kuntz I.D., Rizzo R.C. (2015). DOCK 6: Impact of new features and current docking performance. J. Comput. Chem..

[8] Mobley D.L., Graves A.P., Chodera J.D., McReynolds A.C., Shoichet B.K., Dill K.A. (2007). Predicting absolute ligand binding free energies to a simple model site. J. Mol. Biol..

[9] Chodera J.D., Mobley D.L., Shirts M.R., Dixon R.W., Branson K., Pande V.S. (2011). Alchemical free energy methods for drug discovery: Progress and challenges. Curr. Opin. Struct. Biol..

[10] Abel R., Wang L., Mobley D.L., Friesner R.A. (2017). A critical review of validation, blind testing, and real-world use of alchemical protein–ligand binding free energy calculations. Curr. Top. Med. Chem..

[11] Wang E., Sun H., Wang J., Wang Z., Liu H., Zhang J.Z., Hou T. (2019). End-point binding free energy calculation with MM/PBSA and MM/GBSA: Strategies and applications in drug design. Chem. Rev..

[12] Genheden S., Ryde U. (2015). The MM/PBSA and MM/GBSA methods to estimate ligand-binding affinities. Expert Opin. Drug Discov..

[13] Wang C., Greene D., Xiao L., Qi R., Luo R. (2018). Recent developments and applications of the MMPBSA method. Front. Mol. Biosci..

[14] Hayes J.M., Archontis G. (2012). MM-GB (PB) SA calculations of protein–ligand binding free energies. Molecular Dynamics-Studies of Synthetic and Biological Macromolecules.

[15] Sasmal S., El Khoury L., Mobley D.L. (2020). D3R Grand Challenge 4: Ligand similarity and MM-GBSA-based pose prediction and affinity ranking for BACE-1 inhibitors. J. Comput.-Aided Mol. Des..

[16] Wang Z., Wang X., Li Y., Lei T., Wang E., Li D., Kang Y., Zhu F., Hou T. (2019). farPPI: A webserver for accurate prediction of protein–ligand binding structures for small-molecule PPI inhibitors by MM/PB (GB) SA methods. Bioinformatics.

[17] Forouzesh N., Mishra N. (2021). An Effective MM/GBSA Protocol for Absolute Binding Free Energy Calculations: A Case Study on SARS-CoV-2 Spike Protein and the Human ACE2 Receptor. Molecules.

[18] Sargolzaei M. (2020). Effect of nelfinavir stereoisomers on coronavirus main protease: Molecular docking, molecular dynamics simulation and MM/GBSA study. J. Mol. Graph. Model..

[19] Onufriev A. (2008). Chapter 7—Implicit Solvent Models in Molecular Dynamics Simulations: A Brief Overview. Annu. Rep. Comput. Chem..

[20] Onufriev A.V., Izadi S. (2018). Water models for biomolecular simulations. Wiley Interdiscip. Rev. Comput. Mol. Sci..

[21] Jorgensen W.L., Chandrasekhar J., Madura J.D., Impey R.W., Klein M.L. (1983). Comparison of simple potential functions for simulating liquid water. J. Chem. Phys..

[22] Chen D., Chen Z., Chen C., Geng W., Wei G.W. (2011). MIBPB: A software package for electrostatic analysis. J. Comput. Chem..

[23] Cai Q., Ye X., Wang J., Luo R. (2011). On-the-fly numerical surface integration for finite-difference Poisson–Boltzmann methods. J. Chem. Theory Comput..

[24] Onufriev A., Bashford D., Case D.A. (2000). Modification of the Generalized Born Model Suitable for Macromolecules. J. Phys. Chem. B.

[25] Onufriev A., Bashford D., Case D.A. (2004). Exploring protein native states and large-scale conformational changes with a modified generalized born model. Proteins Struct. Funct. Bioinform..

[26] Onufriev A.V., Case D.A. (2019). Generalized Born implicit solvent models for biomolecules. Annu. Rev. Biophys..

[27] Gohlke H., Kiel C., Case D.A. (2003). Insights into protein–protein binding by binding free energy calculation and free energy decomposition for the Ras–Raf and Ras–RalGDS complexes. J. Mol. Biol..

[28] Wang J. (2020). Fast identification of possible drug treatment of coronavirus disease-19 (COVID-19) through computational drug repurposing study. J. Chem. Inf. Model..

[29] Zhang J., Zhang H., Wu T., Wang Q., van der Spoel D. (2017). Comparison of implicit and explicit solvent models for the calculation of solvation free energy in organic solvents. J. Chem. Theory Comput..

[30] Dzubiella J., Swanson J., McCammon J. (2006). Coupling nonpolar and polar solvation free energies in implicit solvent models. J. Chem. Phys..

[31] Gomes J., Ramsundar B., Feinberg E.N., Pande V.S. (2017). Atomic convolutional networks for predicting protein–ligand binding affinity. arXiv.

[32] Arka D., Anuj K., William W., Jordan R., Vipin K. (2021). Physics-guided Neural Networks (PGNN): An Application in Lake Temperature Modeling. arXiv.

[33] Karpatne A., Atluri G., Faghmous J.H., Steinbach M., Banerjee A., Ganguly A., Shekhar S., Samatova N., Kumar V. (2017). Theory-Guided Data Science: A New Paradigm for Scientific Discovery from Data. IEEE Trans. Knowl. Data Eng..

[34] Li L., Snyder J.C., Pelaschier I.M., Huang J., Niranjan U.N., Duncan P., Rupp M., Müller K.R., Burke K. (2016). Understanding machine-learned density functionals. Int. J. Quantum Chem..

[35] Liu J., Wang K., Ma S., Huang J. (2013). Accounting for linkage disequilibrium in genome-wide association studies: A penalized regression method. Stat. Its Interface.

[36] Muralidhar N., Bu J., Cao Z., He L., Ramakrishnan N., Tafti D., Karpatne A. (2020). Physics-guided deep learning for drag force prediction in dense fluid-particulate systems. Big Data.

[37] Hautier G., Fischer C.C., Jain A., Mueller T., Ceder G. (2010). Finding nature’s missing ternary oxide compounds using machine learning and density functional theory. Chem. Mater..

[38] Fischer C.C., Tibbetts K.J., Morgan D., Ceder G. (2006). Predicting crystal structure by merging data mining with quantum mechanics. Nat. Mater..

[39] Curtarolo S., Hart G.L., Nardelli M.B., Mingo N., Sanvito S., Levy O. (2013). The high-throughput highway to computational materials design. Nat. Mater..

[40] Forouzesh N., Izadi S., Onufriev A.V. (2017). Grid-based surface generalized Born model for calculation of electrostatic binding free energies. J. Chem. Inf. Model..

[41] Izadi S., Harris R.C., Fenley M.O., Onufriev A.V. (2018). Accuracy comparison of generalized Born models in the calculation of electrostatic binding free energies. J. Chem. Theory Comput..

[42] Forouzesh N., Mukhopadhyay A., Watson L.T., Onufriev A.V. (2020). Multidimensional Global Optimization and Robustness Analysis in the Context of Protein-Ligand Binding. J. Chem. Theory Comput..

[43] Izadi S., Aguilar B., Onufriev A.V. (2015). Protein–Ligand Electrostatic Binding Free Energies from Explicit and Implicit Solvation. J. Chem. Theory Comput..

[44] Meng Z., Xia K. (2021). Persistent spectral–based machine learning (PerSpect ML) for protein–ligand binding affinity prediction. Sci. Adv..

[45] Cain S., Risheh A., Forouzesh N. (2021). Calculation of Protein-Ligand Binding Free Energy Using a Physics-Guided Neural Network. Proceedings of the 2021 IEEE International Conference on Bioinformatics and Biomedicine (BIBM), Virtual, 9–12 December 2021.

[46] Sigalov G., Fenley A., Onufriev A. (2006). Analytical Electrostatics for Biomolecules: Beyond the Generalized Born Approximation. J. Chem. Phys..

[47] Still W.C., Tempczyk A., Hawley R.C., Hendrickson T. (1990). Semianalytical Treatment of Solvation for Molecular Mechanics and Dynamics. J. Am. Chem. Soc..

[48] Case D.A., Cheatham T.E., Darden T., Gohlke H., Luo R., Merz K.M., Onufriev A., Simmerling C., Wang B., Woods R.J. (2005). The Amber biomolecular simulation programs. J. Comput. Chem..

[49] Genheden S., Kuhn O., Mikulskis P., Hoffmann D., Ryde U. (2012). The normal-mode entropy in the MM/GBSA method: Effect of system truncation, buffer region, and dielectric constant. J. Chem. Inf. Model..

[50] Numata J., Wan M., Knapp E.W. (2007). Conformational entropy of biomolecules: Beyond the quasi-harmonic approximation. Genome Informatics.

[51] Zhang S., Tong H., Xu J., Maciejewski R. (2019). Graph convolutional networks: A comprehensive review. Comput. Soc. Netw..

[52] Coley C., Barzilay R., Green W., Jaakkola T., Jensen K. (2017). Convolutional Embedding of Attributed Molecular Graphs for Physical Property Prediction. J. Am. Chem. Soc..

[53] Ramsundar B., Eastman P., Walters P., Pande V. (2019). Deep Learning for the Life Sciences: Applying Deep Learning to Genomics, Microscopy, Drug Discovery, and More.

[54] Duvenaud D., Maclaurin D., Aguilera-Iparraguirre J., Gómez-Bombarelli R., Hirzel T., Aspuru-Guzik A., Adams R.P. (2015). Convolutional Networks on Graphs for Learning Molecular Fingerprints. arXiv.

[55] Case D.A., Belfon K., Ben-Shalom I., Brozell S.R., Cerutti D., Cheatham T., Cruzeiro V.W.D., Darden T., Duke R.E., Giambasu G. (2020). Amber 2020.

[56] Luo R., David L., Gilson M.K. (2002). Accelerated Poisson–Boltzmann Calculations for Static and Dynamic Systems. J. Comput. Chem..

[57] Wang J., Luo R. (2010). Assessment of linear finite-difference Poisson–Boltzmann solvers. J. Comput. Chem..

[58] Liu Z., Li Y., Han L., Li J., Liu J., Zhao Z., Nie W., Liu Y., Wang R. (2015). PDB-wide collection of binding data: Current status of the PDBbind database. Bioinformatics.

[59] Wang B., Zhao Z., Nguyen D.D., Wei G.W. (2017). Feature functional theory–Binding predictor (FFT–BP) for the blind prediction of binding free energies. Theor. Chem. Accounts.

[60] Wang J., Wolf R.M., Caldwell J.W., Kollman P.A., Case D.A. (2004). Development and testing of a general amber force field. J. Comput. Chem..

[61] Maier J.A., Martinez C., Kasavajhala K., Wickstrom L., Hauser K.E., Simmerling C. (2015). ff14SB: Improving the accuracy of protein side chain and backbone parameters from ff99SB. J. Chem. Theory Comput..

[62] Ponder J.W., Case D.A. (2003). Force fields for protein simulations. Adv. Protein Chem..

[63] Yin J., Henriksen N.M., Slochower D.R., Shirts M.R., Chiu M.W., Mobley D.L., Gilson M.K. (2017). Overview of the SAMPL5 host–guest challenge: Are we doing better?. J. Comput.-Aided Mol. Des..

[64] Gibb C.L.D., Gibb B.C. (2014). Binding of cyclic carboxylates to octa-acid deep-cavity cavitand. J. Comput.-Aided Mol. Des..

[65] Haiying G., Christopher J.B., Gibb B.C. (2011). Nonmonotonic Assembly of a Deep-Cavity Cavitand. J. Am. Chem. Soc..

[66] Rizzi A., Jensen T., Slochower D.R., Aldeghi M., Gapsys V., Ntekoumes D., Bosisio S., Papadourakis M., Henriksen N.M., De Groot B.L. (2019). The SAMPL6 SAMPLing challenge: Assessing the reliability and efficiency of binding free energy calculations. J. Comput.-Aided Mol. Des..

[67] Xie T., Li Y. (2019). Adding Gaussian Noise to DeepFool for Robustness based on Perturbation Directionality. Aust. J. Intell. Inf. Process. Syst..

